# Effectiveness of Chin Tuck on Laryngeal Penetration: Quantitative Assessment

**DOI:** 10.1007/s00455-020-10238-4

**Published:** 2021-01-05

**Authors:** Joo Young Ko, Dae Youp Shin, Tae Uk Kim, Seo Young Kim, Jung Keun Hyun, Seong Jae Lee

**Affiliations:** 1 Department of Rehabilitation Medicine, College of Medicine, Dankook University, 119 Dandae-ro, Dongnam-gu, Cheonan, Chungnam 31116 Republic of Korea; 2 Department of Nanobiomedical Science & BK21 PLUS NBM Research Center for Regenerative Medicine, Dankook University, 119 Dandae-ro, Dongnam-gu, Cheonan, Chungnam 31116 Republic of Korea; 3 Institute of Tissue Regeneration Engineering (ITREN), Dankook University, 119 Dandae-ro, Dongnam-gu, Cheonan, Chungnam 31116 Republic of Korea

**Keywords:** Deglutition disorders, Dysphagia, Chin tuck, Effectiveness, Prevention, Videofluoroscopy

## Abstract

The effectiveness of the chin tuck maneuver is still controversial, despite being widely used in clinical practice. The chin tuck maneuver has been shown to be able to reduce or eliminate aspiration in a group of patients with a number of favorable conditions, but its effectiveness in preventing or managing penetration remains unclear. This study was designed to investigate whether the chin tuck maneuver is effective in reducing penetration. Images from a videofluoroscopic swallowing study (VFSS) taken from 76 patients with penetration were collected and reviewed retrospectively. The severity of penetration was assessed by the penetration ratio (ratio of the penetration depth to the length of the epiglottis) measured and calculated from the images in which the deepest penetration was observed. The penetration ratio was significantly decreased in the chin tuck posture compared with the ratio in the neutral position (*p* = 0.001). Significant reducing effect was observed in 26 (34.2%) out of 76 patients. When comparing other parameters of VFSS, residues in the vallecular and pyriformis sinuses were less severe in the effective group. Chin tuck significantly decreased residues in both effective and ineffective group. The results demonstrate that the chin tuck maneuver can reduce penetration, but its effectiveness is limited.

## Introduction

Dysphagia is a common disorder that can occur as a result of various neurological disorders, such as cerebrovascular accidents, head injuries, and degenerative diseases [1]. Protection of the laryngeal airway, which is crucial for safe and efficient swallowing, is frequently impaired in dysphagia, leading to invasion of food material or liquid into the larynx [2]. Such conditions may cause various complications, including respiratory morbidity or even death [3]. Invasion limited to above the vocal folds is called penetration, while aspiration means that the invaded bolus enters the deeper airway below the vocal folds [1]. Penetration has not received much attention from researchers because it is a milder form of airway invasion that can occur even in about 10% of normal adults [4]. However, penetration can still be hazardous to pulmonary health especially when it is repeated and goes deeper in the laryngeal vestibule [5]. In a previous report, approximately two-thirds of patients with aspiration pneumonia showed laryngeal penetration [6]. In other study that correlated VFSS (videofluoroscopic swallowing study) findings and clinical data, patients with laryngeal penetration were four times more likely to have pneumonia within 6 months before or after VFSS examination [7]. Considering those reports, it is believed that the clinicians should pay more attention to prevention or reduction of penetration when taking care of the patients with dysphagia.

Treatment of dysphagia has traditionally been differentiated into restorative and compensatory strategies [8]. Restorative therapy is centered on accelerating the recovery process, and compensatory techniques are centered on keeping patients safe when swallowing [9]. Among the compensatory techniques, chin tuck (also known as chin down or neck flexion) is one of the methods most widely used to prevent instances of airway invasion of food material such as penetration or aspiration [3]. With chin tuck, airway protection can be improved by posterior shift of the anterior pharyngeal structures, which narrows the laryngeal entrance by shortening the distance from epiglottis to the pharyngeal wall and widening the angle of the epiglottis to the anterior wall of the trachea [1, 10, 11]. Patients with delayed swallowing trigger have been demonstrated to benefit of preventing aspiration from this maneuver [12]. However, despite its clinical popularity, it was found that the chin tuck eliminates aspiration in only 8–50% of cases with neurologic disorders in previous studies [13–15]. It was also suggested that the chin tuck can prevent aspiration in the context of a few favorable conditions, including female sex, absence of residue in the pyriform sinus, delayed swallowing trigger, reduced laryngeal elevation, and penetration of thin liquid [12].

Although a number of studies have been performed to explore the effectiveness of the chin tuck maneuver, most of them investigated the effectiveness by observing the presence or elimination of aspiration, rather than measuring its severity or depth [9, 12–14]. Only one study included the patients with penetration, in which the presence of effect was defined as decrease in more than or equal to 1.0 of 8-point penetration-aspiration scale (PAS) [3]. However, PAS is only a semi-quantitative scale measuring the depth of penetration and aspiration from the videofluoroscopic swallowing study (VFSS) and the score 2–5 corresponds to penetration [16]. In PAS, penetration is graded according to removal and contact with vocal cords, rather than measuring the depth of penetration precisely [16]. It seems that more objective and quantitative assessment is required to verify whether the chin tuck is effective in preventing or reducing penetration that may increase the risk of pneumonia [6, 7]. Currently, the videofluoroscopic swallowing study (VFSS) is regarded as a standard evaluation tool that permits direct visualization and objective evaluation of bolus transfer and swallowing events [15]. The aims of this study are to reveal the effect of the chin tuck maneuver on the severity of penetration by quantitating the depth of penetration from the images of VFSS and to identify the factors affecting the efficacy of chin tuck maneuver.

## Materials and Methods

### Materials

VFSS video files taken from 76 patients who were referred or admitted for performing VFSS due to dysphagia symptoms such as drooling and poor oral management, food and/or liquids leaking from the nasal cavity, globus sensation in the neck, complaints of pain when swallowing, wet or gurgly sounding voice during or after eating or drinking, and coughing during or right after eating or drinking between October 2016 and July 2018 were selected retrospectively from the storage of Dankook University Hospital. The files were included in the analysis when (1) the penetration of the bolus mixed with contrast media into the laryngeal vestibule was evident and (2) the study was performed in both neutral and chin tuck positions. Files were excluded when they (1) showed aspiration in VFSS or (2) when the chin tuck posture was not performed properly. The causes of dysphagia are presented in Table 1 and the pathophysiology of dysphagia is presented in Table 2.

**Table 1:** Etiologic distribution of the patients from whom the VFSS was taken

Etiology	Number of patients
Chronic subdural hemorrhage	1
Parkinson's disease	3
Stroke	31
Idiopathic	25
Polymyositis	1
Traumatic brain injury	8
Epilepsy	1
Meningitis	1
Amyotrophic lateral sclerosis	1
Dermatomyositis	1
Hypoxic brain damage	1
Laryngeal cancer	1
Tonsillar cancer	1

Values are the number of subjects

**Table 2:** Pathophysiology of dysphagia

Etiology	Number of patients
Incomplete lip closure	26
Delayed oral phase	49
Excessive oral cavity residue	26
Inadequate bolus formation	58
Delayed swallowing reflex	57
Decreased laryngeal elevation	59
Excessive vallecular residue	49
Excessive pyriform sinus residue	41
Exist of pharyngeal wall coating	57

Values are number of subjects

All the VFSS video images were recorded following the protocol described by Logemann [1] with minor modifications. Each patient was instructed to swallow 3-ml boluses of various consistencies mixed with contrast medium in a seated position, while the video images were recorded with lateral projection and stored digitally at a speed of 30 frames per second. The order of the consistencies swallowed was swallowing of thick liquid (water-soluble barium sulfate diluted to 70%) (IDDSI—International Dysphagia Diet Standardization Initiative score 3) three times, swallowing of rice porridge (IDDSI score 2) two times, swallowing of curd-type yogurt (IDDSI score 1) two times, and swallowing of thin liquid (water-soluble barium sulfate diluted to 35%) (IDDSI score 0) three times, followed by drinking of 5 ml of thin liquid from a cup two times. Whenever penetration appeared during a swallowing in neutral position, the patients were requested to perform the chin tuck maneuver (guided to “tuck your chin as close to your sternum as possible,” as described in a previous study [10]) and the swallowing was repeated with the same consistency and volume. If chin tuck posture seemed not to be proper, the patients were requested to perform the chin tuck maneuver once again.

## Methods

### Assessment of the Chin Tuck Effect

**Fig. 1:**
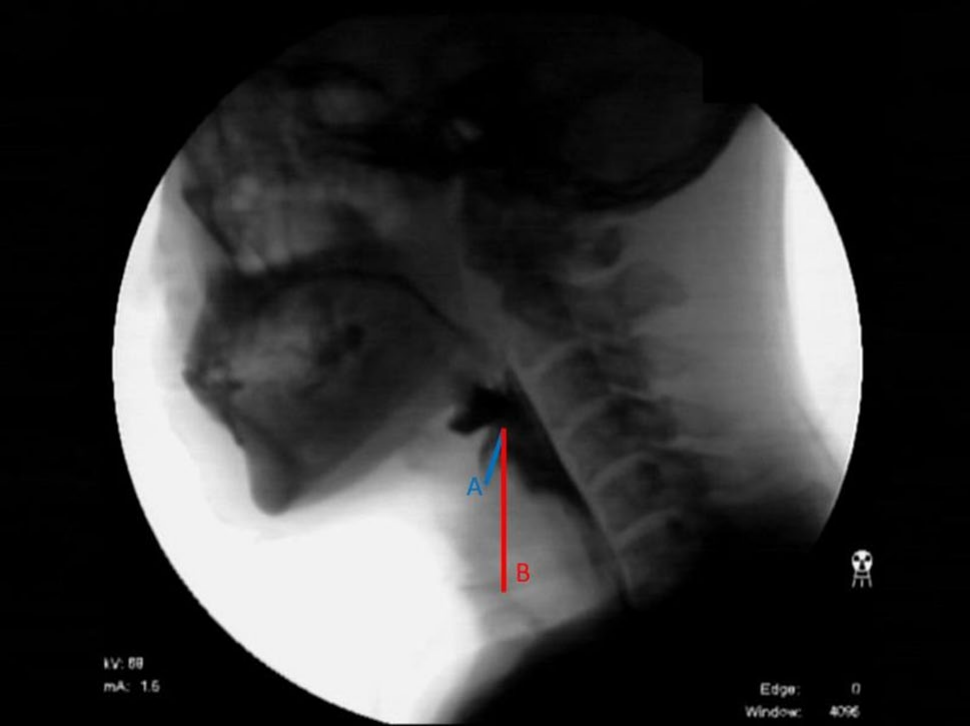
Measurement of the penetration depth and the epiglottis length. Blue-colored line **a** denotes the penetration depth that was measured as the straight length from the tip of the epiglottis to the endpoint of penetration. Red-colored line **b** indicates the epiglottis length that was measured as the straight length from the tip of the epiglottis to the anterior tip of the true vocal folds

Full length VFSS video images were reviewed frame by frame by a physiatrist experienced in VFSS analysis. Every effort was made to exclude the effect of the possibilities other than chin tuck, including the events of dry swallow or throat clear in previous swallow. When the residue was present in the laryngeal vestibule, a new passage of bolus was distinguished carefully. A frame image showing the deepest penetration was selected from all images recorded in the VFSS session, in which contrast exists deepest between the laryngeal inlet and vocal fold regardless of bolus texture and order of swallow. The penetration depth was measured in that frame image as the straight length from the tip of the epiglottis to the endpoint of penetration. The epiglottis length was measured as the straight length from the tip of the epiglottis to the foremost end of true vocal folds (Fig. 1), equivalent to a point at which the superior margin of vocal fold shadow meets the anterior wall of larynx. The penetration ratio was also determined in that image, which was defined as the ratio of penetration depth to epiglottis length. ImageJ® software by NIH was used for all length measurements. To confirm the validity of the length measurement described above, intra- and inter-rater reliability for two raters were tested and the results showed excellent agreement of intraclass correlation coefficient ranging from 0.828 to 0.998 in both neutral and chin tuck position.

The penetration ratio was compared between the neutral and chin tuck positions. The penetration ratio was graded into 5 levels according to the severity of penetration at the interval of 0.2 (grade 1 (very shallow): less than 0.2; grade 2 (shallow): from 0.2 to less than 0.4; grade 3 (moderate): from 0.4 to less than 0.6; grade 4 (deep): from 0.6 to less than 0.8; grade 5 (very deep): from 0.8 to 1.0). The chin tuck maneuver was considered effective when significant reduction was observed, which was defined as more than or equal to 1-grade reduction in chin tuck position.

### Other VFSS Parameters

VFSS parameters associated with swallowing process suggested by Logemann and previous investigators other than penetration were also evaluated for the oral and pharyngeal phases (Table 3) [1, 17–19]. These parameters have been proposed in previous studies which evaluated the effectiveness of the chin tuck maneuver [3, 9, 12]. In the oral phase, completeness of lip closure, presence of oral residue, and presence of premature bolus leakage were assessed, and the oral transit time (OTT) was measured as the time elapsed from the backward movement of the bolus until the bolus head reached the lower edge of the mandible [1]. In the pharyngeal phase, delayed triggering of pharyngeal swallowing, height of laryngeal elevation, and the presence of residues in the valleculae and pyriformis sinuses were assessed, and the pharyngeal delayed time (PDT) and pharyngeal transit time (PTT) were measured. Residues in valleculae and pyriformis sinuses defined as clearly visualized barium lining in the valleculae and pyriformis sinuses after swallowing were graded into 4 levels (none (1); minimal (2), < 10% of bolus; moderate (3), from 10 to 50% of bolus; maximal (4), > 50% of bolus) [18]. PDT was defined as the time from the arrival of the bolus at the lower edge of the mandible until pharyngeal swallow was triggered [1], and PTT was defined as the time from the arrival of the bolus head at the lower edge of the mandible until the bolus tail passed through the cricopharyngeal region [1]. All parameters were measured in upright position after the first swallowing of a bolus in the consistency which showed deepest penetration and re-evaluated in chin tuck posture. The results were compared between the patients whose chin tuck was effective and ineffective according to the classification described in previous section.

**Table 3:** Details of VFSS parameters associated with swallowing process

Parameter of swallowing process	Definition	Interpretation
Oral phase		
Lip closure	Labial seal to ensure that no food or liquid falls from the mouth	Complete/incomplete
Bolus formation	Mastication and preparation of a semicohesive bolus or ball	Adequate/inadequate
Oral cavity residue	Barium residue exists on the floor of the mouth, tongue, hard palate, and anterolateral sulcus	None/exist
Pharyngeal phase		
Triggering of pharyngeal swallow	The timing of triggering of pharyngeal swallow (Elevation and anterior movement of hyoid and larynx with bolus head passing the spot where the lower edge of the mandible crosses the tongue base)	Normal (< 0.5 s)/delayed (≥ 0.5 s)
Laryngeal elevation	Getting closer and tilting forward of the arytenoids to the base of epiglottis and closing the airway as larynx elevates	Intact (≥ 2 cm)/Decreased (< 2 cm)
Residue in vallecula and pyriform sinus	Barium residue on vallecula and pyriform sinus after swallowing	Grade 0: none; Grade 1: < 10% of bolus: grade 2: 10% to 50% of bolus: grade 3: > 50% of bolus
Pharyngeal wall coating	Barium is coated the pharyngeal wall after swallowing	None/exist

The definitions of parameters are referenced to Logemann [1]. The grading of residue in vallecula and pyriform sinus is based on the study by Han et al. [18]

### Statistical Analysis and Study Approval

The study protocol was approved by the Institutional Review Board of Dankook University Hospital (IRB No. 2019-10-006). Statistical analyses were carried out using SPSS for Windows version 25.0 (SPSS, Chicago, IL, USA). Categorical parameters were compared by Fisher’s exact test, and within-group changes were verified by paired *t *test. Comparison of numerical data between the groups was performed by independent *t* test, except for ordinal variables for which the Mann–Whitney *U* test was used. Correlation between food consistency and 8PPAS was analyzed by Spearman’s rank correlation. Inter- and intra-rater reliability were calculated by intraclass correlation coefficient. Significance was accepted for *p* values < 0.05.

## Results

### Change in Penetration Depth and Ratio

**Fig. 2:**
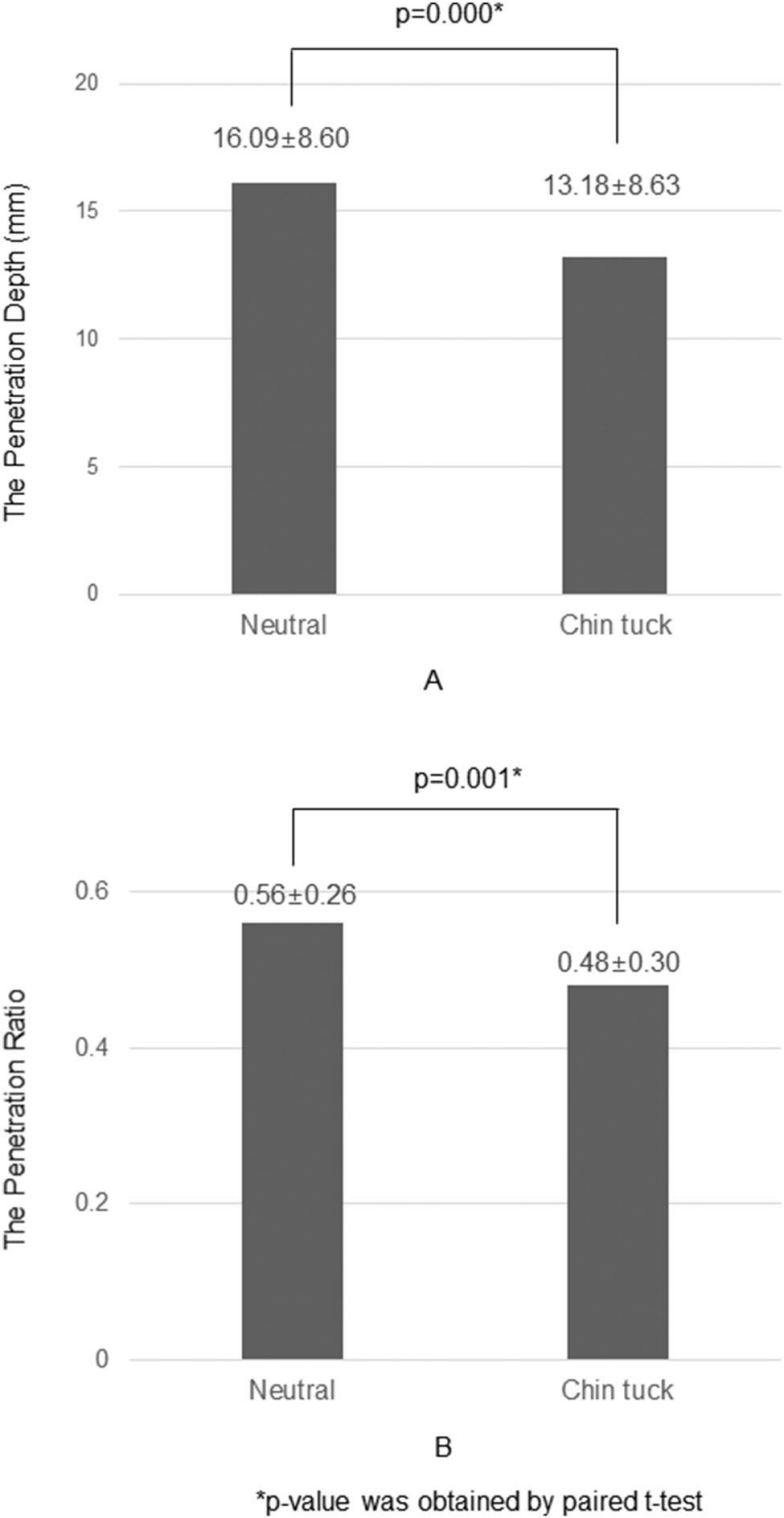
Change in the penetration depth and the penetration ratio. Chart **a** denotes change in the penetration depth and chart **b** denotes change in the penetration ratio

The penetration depth was 16.09 ± 8.60 mm in the neutral position and 13.18 ± 8.63 mm in the chin tuck position. Decrease of the penetration depth in the chin tuck position was statistically significant (*p* = 0.000) (Fig. 2). The penetration ratio was 0.56 ± 0.26 in the neutral position and 0.48 ± 0.30 in the chin tuck position; the ratio also decreased significantly in the chin tuck posture compared with that in the neutral position (*p* = 0.001)(Fig. 2).

### Food Consistency and 8-Point Penetration-Aspiration Scale (8PPAS)

The correlation analysis revealed significant relationship between food consistency and the 8-point penetration-aspiration scale (8PPAS) (*ρ* = 0.335, *p* = 0.000). Mean values of the 8PPAS of each food consistency are presented in Table 4.

**Table 4:** Severity of penetration according to the food consistencies

Food consistency	8PPAS	Ineffective group (*n* = 50)	Effective group (*n* = 26)
Thick liquid	1.74 ± 1.25	2.04 ± 1.62	1.27 ± 0.53
Rice porridge	1.85 ± 1.12	2.26 ± 1.69	1.48 ± 1.42*
Curd-type yogurt	1.92 ± 1.36	2.36 ± 1.79	1.32 ± 0.85*
Thin liquid	2.81 ± 1.52	3.65 ± 2.40	2.38 ± 1.65*
Drinking from a cup	2.85 ± 1.37	3.30 ± 1.85	2.88 ± 2.06

Values are the mean ± standard deviation; 8PPAS: 8-point penetration-aspiration scale

*p < 0.05 by Mann–Whitney *U* test between effective and ineffective groups

### Comparison Between Effective and Ineffective Groups

Chin tuck was effective in 26 out of 76 (34.2%) patients with penetration. There is no significant difference in age distribution between effective and ineffective group (65.62 ± 17.66 years vs 68.22 ± 12.19 years). Gender distribution also showed no significant difference between effective (male:female = 14:12) and ineffective group (male:female = 29:21). Mean values of the 8PPAS were higher in the ineffective group at all food consistencies except for swallowing thick liquid and drinking thin liquid from a cup (Table 4). In both neutral and chin tuck position, effective group showed significantly less amount of residues in the vallecular and pyriformis sinuses (Table 5). However, the residues decreased in chin tuck position in both effective and ineffective groups (Table 5). None of the other parameters showed significant differences between neutral and chin tuck position and between effective and ineffective group.

**Table 5:** Results of other VFSS parameters in effective and ineffective group

Parameters			Ineffective group (*n* = 50)	Effective group (*n* = 26)	*p* value
Time parameters (sec)					
OTT	Neutral		3.41 ± 6.11	4.01 ± 6.08	0.687^‡^
	Chin tuck		2.61 ± 3.54	2.93 ± 4.73	0.734^‡^
	*p* value		0.177**	0.092**	
PDT	Neutral		1.60 ± 3.42	0.73 ± 0.81	0.206^‡^
	Chin tuck		1.58 ± 2.61	0.83 ± 1.49	0.178^‡^
	*p* value		0.971**	0.726**	
PTT	Neutral		2.27 ± 3.41	1.27 ± 0.80	0.148^‡^
	Chin tuck		2.10 ± 2.59	1.25 ± 1.50	0.128^‡^
	*p* value		0.746**	0.935**	
Residue (grade)					
Vallecular residue	Neutral	1	7	7	0.017*
		2	21	14	
		3	10	3	
		4	12	2	
	Chin tuck	1	10	10	0.008*
		2	23	13	
		3	7	2	
		4	10	1	
	*p* value		0.024**	0.031**	
Pyriform sinus residue	Neutral	1	13	13	0.049*
		2	19	7	
		3	7	4	
		4	11	2	
	Chin tuck	1	18	17	0.037*
		2	17	4	
		3	7	4	
		4	8	1	
	*p* value		0.010**	0.031**	
Other parameters					
Lip closure	Neutral	Complete	47	26	0.279^†^
		Incomplete	3	0	
	Chin tuck	Complete	47	26	0.279^†^
		Incomplete	3	0	
	*p* value		1.000***	1.000***	
Oral cavity residue	Neutral	None	43	25	0.166^†^
		Exist	7	1	
	Chin tuck	None	44	25	0.235^†^
		Exist	6	1	
	*p* value		1.000***	1.000***	
Bolus formation	Neutral	Adequate	25	11	0.347^†^
		Inadequate	25	15	
	Chin tuck	Adequate	29	10	0.084^†^
		Inadequate	21	16	
	*p* value		0.289***	1.000***	
Swallowing reflex	Neutral	Normal	21	14	0.229^†^
		Delayed	29	12	
	Chin tuck	Normal	19	15	0.082^†^
		Delayed	31	11	
	*p* value		0.774***	1.000***	
Laryngeal elevation	Neutral	Intact	36	18	0.501^†^
		Decreased	14	8	
	Chin tuck	Intact	36	21	0.292^†^
		Decreased	14	5	
	*p* value		1.000***	0.250***	
Pharyngeal wall coating	Neutral	None	24	19	0.051^†^
		Exist	26	7	
	Chin tuck	None	28	20	0.060^†^
		Exist	22	6	
	*p* value		0.219***	1.000***	

Values are the mean ± standard deviation for age, time parameters, and residue; otherwise, values are the number of patients

*OTT* oral transit time, *PDT* pharyngeal delayed time, *PTT* pharyngeal transit time

*p* values were obtained from ^†^Fisher’s exact test, ^‡^Independent *t* test, *Mann–Whitney *U* test, **Paired *t* test, and ***McNemar test

## Discussion

Previous studies reported that laryngeal penetration is associated with the risk of aspiration pneumonia when it is found in VFSS [6, 7]. Moreover, deep penetration has been reported to be a predictor of aspiration at least in children [5]. Risk of complication may not be great in penetration, because bolus does not enter airway beyond vocal cords. There is a possibility that VFSS failed to find aspiration or penetration progressed to aspiration after examination in those studies. Even if it is true, occurrence of penetration may suggest a risk of more severe form of airway invasion in the context of swallowing safety. Therefore, clinicians should pay attention to management of penetration especially when the patient shows symptoms related to dysphagia. Behavioral treatments, including chin tuck, have been demonstrated to prevent aspiration in approximately half of patients with dysphagia [20], but their effect on penetration is still not clear. The present study tried to investigate whether the chin tuck can prevent or reduce the laryngeal penetration in the patients with swallowing difficulties.

Most of the studies related to the effectiveness of chin tuck-used PAS, a semi-quantitative ordinal scale that is most commonly used to determine the severity of penetration and aspiration [16]. The depth of airway invasion can be graded by PAS, according to the location and remnant of food material mixed with contrast media, which is visualized in VFSS images [18]. However, PAS is basically an interval scale that is still insufficient to reflect subtle changes in penetration, especially when it is within the intervals between grades. Penetration depth was measured directly from VFSS images in the present study, rather than being scaled, to overcome this limitation of PAS. To eliminate errors from individual variations in size, the ratio of the penetration depth to the length of the epiglottis, namely, the penetration ratio, was calculated. The authors believe that the penetration ratio can reflect the change in penetration depth more precisely than PAS. Consequently, it is also believed that this study demonstrated the effectiveness of chin tuck more accurately than most previous studies in which the chin tuck maneuver was considered effective only when penetration or aspiration was prevented [9, 12–15]. This method has never been attempted previously to the authors’ knowledge. The reliability of this new method also has been tested and the results showed excellent intra- and inter-rater agreement. However, the depth and length measurement, inevitably the ratio calculation, used in the present study can be affected by the position of the epiglottis. They could be measured consistently because all subjects showed penetration during swallowing before inversion of epiglottis. It should be verified whether their measurement is still reliable when the penetration occurs before or after swallow or when the epiglottis is inverted.

The severity of penetration, represented by the penetration ratio, was decreased as a result of the chin tuck maneuver but only by a very small amount in this study. Moreover, a decrease was found in only approximately one-third of the subjects. These results coincide with previous studies regarding the effectiveness of chin tuck for the amelioration of aspiration [13–15]. Chin tuck has been used almost routinely for managing airway invasion in patients with dysphagia, but related studies including the authors’ suggest that clinicians should be cautious in conducting such customary practice and monitor the efficacy in individual patients. If possible, instrumental examination is warranted which can show the effect objectively.

In the comparison between the effective and ineffective groups, the former group showed significantly less amount of residue accumulation in the vallecula and pyriform sinuses, in both neutral and chin tuck position. None of the other parameters of VFSS showed any difference between the groups. Chin tuck decreased the severity of residue in both groups, but the underlying mechanism is not clear. The preceding studies also demonstrated conflicting results about the effect of chin tuck on the pharyngeal contraction [21, 22]. It is unlikely that the chin tuck effect appeared by reducing the residue because both groups showed decrease. However, the results suggest that the patients with less amount of residue in valleculae and pharynx may be better candidates for chin tuck. Although a number of conditions, such as delay in the swallowing trigger, reduced laryngeal elevation and penetration of thin liquids [12], and female sex [3], were previously proposed to favor the effectiveness of chin tuck, the effectiveness of chin tuck was not affected by those conditions in this study. It is speculated that these factors may not influence the effectiveness of chin tuck in patents with penetration only.

This study has some limitations. First, the depth of penetration and length of the epiglottis were measured in straight lines despite their curved forms. The straight distance may not be identical to the true length of penetration, but it would be reasonable to think that they are proportional to each other. Second, only the immediate effect of the chin tuck maneuver was measured, and the effectiveness of this maneuver after enough training still needs further research. As a compensatory technique, presence of long-term effect is still controversial. Previous studies revealed that the duration of laryngeal vestibule closure increases in chin tuck position, but returns to baseline after resuming neutral position [23, 24]. However, repetition and training may affect the effectiveness because proper positioning is a key to benefit from chin tuck [3]. Prevention of possible later complications also need to be investigated in further researches. Third, patients with insufficient chin tuck were excluded, but the neck flexion angle was not measured exactly, making the results vulnerable to possible selection bias. Changes in cervical vertebral curvature, which is especially common in elderly patients, are likely to make it difficult to maintain the proper chin tuck posture, further limiting its effectiveness. Fourth, since all penetration occurred during swallow before inversion of epiglottis in the present study, the measuring method may not be used when the penetration occurs in other timing. Lastly, effect of the timing of penetration on the effectiveness of chin tuck could not be analyzed because all penetrations occurred during swallow.

## Conclusions

The results suggest that the chin tuck maneuver is less effective than expected. Laryngeal penetration was reduced by the chin tuck maneuver, but significant reduction was observed in only one-third of the patients. The clinical usefulness of the chin tuck maneuver is limited especially for eliminating or reducing penetration, and it is preferable to confirm the effectiveness prior to prescribing.
